# The Role of Dendritic Cells in Adaptive Immune Response Induced by OVA/PDDA Nanoparticles

**DOI:** 10.3390/vaccines13010076

**Published:** 2025-01-16

**Authors:** Daniele R. Pereira, Yunys Pérez-Betancourt, Bianca C. L. F. Távora, Geraldo S. Magalhães, Ana Maria Carmona-Ribeiro, Eliana L. Faquim-Mauro

**Affiliations:** 1 Laboratory of Immunopathology, Butantan Institute, São Paulo 05585-000, Brazil; rodriguesdaniele663@gmail.com (D.R.P.); bianca.tavora@fundacaobutantan.org.br (B.C.L.F.T.); geraldo.magalhaes@butantan.gov.br (G.S.M.); 2 Department of Immunology, Institute of Biomedical Sciences, University of São Paulo, São Paulo 04021-001, Brazil; 3 Department of Biochemistry, Chemistry Institute, University of São Paulo, São Paulo 04021-001, Brazil; ybetancourt@bsd.uchicago.edu (Y.P.-B.); mcribeir@iq.usp.br (A.M.C.-R.)

**Keywords:** nanoparticles, poly-diallyl-dimethyl-ammonium chloride (PDDA), dendritic cell, adjuvant, immune response, vaccine

## Abstract

**Background/Objective**: Cationic polymers were shown to assemble with negatively charged proteins yielding nanoparticles (NPs). Poly-diallyl-dimethyl-ammonium chloride (PDDA) combined with ovalbumin (OVA) yielded a stable colloidal dispersion (OVA/PDDA-NPs) eliciting significant anti-OVA immune response. Dendritic cells (DCs), as sentinels of foreign antigens, exert a crucial role in the antigen-specific immune response. Here, we aimed to evaluate the involvement of DCs in the immune response induced by OVA/PDDA. **Methods**: In vivo experiments were used to assess the ability of OVA/PDDA-NPs to induce anti-OVA antibodies by ELISA, as well as plasma cells and memory B cells using flow cytometry. Additionally, DC migration to draining lymph nodes following OVA/PDDA-NP immunization was evaluated by flow cytometry. In vitro experiments using bone marrow-derived DCs (BM-DCs) were used to analyze the binding and uptake of OVA/PDDA-NPs, DC maturation status, and their antigen-presenting capacity. **Results:** Our data confirmed the potent effect of OVA/PDDA-NPs inducing anti-OVA IgG1 and IgG2a antibodies with increased CD19^+^CD138^+^ plasma cells and CD19^+^CD38^+^CD27^+^ memory cells in immunized mice. OVA/PDDA-NPs induced DC maturation and migration to draining lymph nodes. The in vitro results showed higher binding and the uptake of OVA/PDDA-NPs by BM-DCs. In addition, the NPs were able to induce the upregulation of costimulatory and MHC-II molecules on DCs, as well as TNF-α and IL-12 production. Higher OVA-specific T cell proliferation was promoted by BM-DCs incubated with OVA/PDDA-NPs. **Conclusions**: The data showed the central role of DCs in the induction of antigen-specific immune response by OVA-PDDA-NPs, thus proving that these NPs are a potent adjuvant for subunit vaccine design.

## 1. Introduction

The induction of protective immune responses against different pathogens, including different viruses and cancer, has been a difficult challenge for the scientific community [1,2,3,4]. In this sense, distinct strategies have been designed to promote the appropriate immune system activation with the goal of inducing enhanced memory immunity to protect against future pathogen infections [2]. Neutralizing antibodies produced by B cells and T lymphocyte responses mediate the mechanisms of protection against pathogens induced by vaccines [3]. In the induction of protective response against distinct pathogens, subsets of CD4^+^ T cells provide help for B cell development and antibody production in lymphoid tissues [5] and the generation of memory immunity. CD8^+^ T cell response is also induced during the immune system activation by infection or vaccination, and CD8^+^ T memory cells are generated in these processes [6]. Therefore, increased numbers of experimental vaccines have been focused on creating molecular networks to initiate and activate innate and adaptive immune responses [7]. Distinct molecular approaches have been studied to improve innate immune cell activation by creating local inflammatory niches, followed by lymph node delivery and the induction of robust humoral and cellular response. As known, some vaccines based on antigen/pathogen subunits induce a modest immunogenic response, which justifies the use of adjuvant molecules [3] as a tool to increase antigen-specific immune response in quantitative and qualitative terms [4].

Among different mechanisms mediated by adjuvants, an important role is the ability to activate innate immune cells, mainly dendritic cells (DCs). This stimulation promotes increasing antigen uptake, cytokines production, and the expression of costimulatory molecules on the DC membrane surface, which are effective in activating and differentiate T cells [8,9,10,11]. DCs and other cells of the innate immune system can become activated not only by distinct pathogen-associated molecular patterns (PAMPs) via pattern recognition receptors (PRRs) but also by tissue damage that results in the release of damage-associated molecular patterns (DAMPs) [12,13,14]. Therefore, the DC population exerts a critical role in the recognition of antigens, followed by migration to the lymphoid tissue and activation of T cells. DCs interact with T cells by mediating three signs: 1–processed antigen presentation in MHC complexes to T lymphocytes through T cell receptor (TCR) signaling; 2–the expression of costimulatory molecules in the cellular membrane for interacting with ligands expressed in the T cell membrane; and 3–the production of cytokines as mediators of T cell effector polarization [15,16,17].

Aluminum salts are the most common adjuvants licensed [18]. Concerning the growth of nanotechnology research, nanostructures are widely studied for vaccine design, mainly as adjuvant tools [12]. Nanoparticles such as liposomes, microspheres, chitosan-nanoparticles, and polymers have been evaluated as potent antigen carriers capable of immune-adjuvant activity [14,15,19,20,21]. The immunomodulatory properties of cationic polymers such as chitosan and poly (ethylene imine) (PEI) have been investigated, aiming at vaccine development. Carroll et al. (2016) showed the ability of chitosan to induce BM-DC maturation in an IFN-I-dependent manner, consequently providing cyclic GMP-AMP synthase (c-GAS) and stimulator of interferon genes (STING)-dependent Th1 immunity [22]. The adjuvanticity of PEI and PEI-based particles has been correlated with the ability to generate danger signals that mediate the immune cells’ activation as APCs and the induction of adaptive responses [23]. Pérez-Betancourt and collaborators (2020) demonstrated that poly (diallyl dimethyl ammonium chloride) (PDDA) conjugated to OVA (OVA/PDDA-NPs) was 170 nm in hydrodynamic diameter and displayed high colloidal stability, low polydispersity, and positive zeta potential (+30 mV), inducing a robust anti-OVA IgG1 and IgG2a antibody production, as well as T cell response in immunized mice, as verified by the immunization with OVA and Alum [24]. The present data further reinforce the important role that cationic polymers can have in vaccine design. Herein, the in vitro and in vivo experiments show that OVA/PDDA-NPs induced a potent anti-OVA antibody response, as previously demonstrated by Pérez-Betancourt et al. (2020) [24]. Furthermore, higher numbers of plasma cells and memory B cells were also verified in mice immunized with OVA/PDDA-NPs compared with OVA/Alum immunization. OVA/PDDA-NPs induced DC maturation and also migration to draining lymph nodes. The higher uptake of OVA/PDDA-NPs compared with soluble OVA by immature DCs was also verified, resulting in T cell response. DCs incubated in vitro with OVA/PDDA-NPs were also able to significantly improve OVA-specific CD4^+^T cell proliferation. Taken together, OVA/PDDA-NPs exert a direct effect on DCs, leading to their maturation and the consequent development of CD4^+^T cell response that potentiates antibody production and the generation of memory B cells.

## 2. Materials and Methods

### 2.1. Antigens and Adjuvants

Ovalbumin grade V (OVA) (Sigma-Aldrich, Merck Corporation, Darmstadt, Germany) was used in the in vivo and in vitro experiments. For the in vitro protocols, lipopolysaccharide (LPS, *E. coli*) (Sigma-Aldrich, Merck Corporation, Darmstadt, Germany ) was used in association with OVA. Aluminum hydroxide (Alum) (Sanofi, São Paulo, Brazil) was adsorbed to OVA and used in the in vivo immunization of mice. OVA/PDDA-NP formulation was confirmed by the measurement of turbidity of the dispersion, monitored in a Beckman spectrophotometer at 300 nm, and used in in vitro and in vivo protocols. OVA-FITC (Invitrogen, Thermo Fisher Scientific, Waltham, MA, USA) OVA-FITC conjugated to PDDA (OVA-FITC/PDDA) was used in in vitro experiments.

### 2.2. Experimental Groups

For experimental procedures, isogenic BALB/c male mice (7–8 weeks) were obtained from Central Animal Facility (Butantan Institute, São Paulo, Brazil), and DO11.10 TCR transgenic mice specific for MHC-II–restricted OVA peptide (7–8 weeks) were obtained from Laboratory of Animal Facility (ICB/USP). The BALB/c mice were maintained in the Immunopathology Laboratory facility, while DO11.10 mice were maintained in the Special Laboratory of Pain and Signaling of Butantan Institute under controlled temperatures, 12/12 light/dark ratio, and standard feed and water ad libitum. The current study was conducted after ethical committee approval (CEUAIB: 1282081119).

### 2.3. Endotoxin Removal in OVA and Protein Concentration Experiment

Samples of OVA diluted in water (25 mg/mL) were submitted to a polymyxin column immobilized on Sepharose (Thermo Scientific, Waltham, MA, USA) to remove possible LPS contamination. Afterwards, the protein content was determined by the BCA bicinchoninic acid method (Sigma-Aldrich).

### 2.4. Preparation of PDDA/OVA Dispersions

Stock solutions of OVA (10 mg/mL) and PDDA (1 mg/mL) were used to obtain the OVA/PDDA-NPs. Therefore, the NPs were prepared using 100 μg/mL of OVA with 10 μg/mL of PDDA in deionized water, as previously shown by Pérez-Betancourt et al. (2020) [24]. After 30 min, OVA/PDDA-NPs formation was monitored by turbidity analysis by measuring the optical density at 300 nm in a spectrophotometer (Beckman Coulter, Brea, CA, USA). For the experiments, only dispersions with optical density above 0.300 were used in the experiments.

### 2.5. Confirmation of the Mean Hydrodynamic Diameter of NPs by Dynamic Light Scanning (DLS) and Zeta Potential

The average hydrodynamic diameter of the NPs together with the evaluation of the polydispersion index (PdI) was performed by dynamic light scattering (DLS), and evaluation of Zeta-potential was performed using 2 mL of sample in the Zeta Plus Zeta Potential Analyzer equipment.

### 2.6. Ultracentrifugation of OVA/PDDA-NPs

Samples of OVA/PDDA-NPs prepared as previously described and soluble OVA (100 μg/mL) were centrifuged at 14,500 rpm for 30 min. After that, the supernatants (SBNs) were collected, and the protein content was determined by BCA.

### 2.7. Immunization Protocol

Groups of BALB/c male mice (aged 7–8 weeks) were immunized with 200 μL of OVA/PDDA-NPs (100 μg/10 μg/mL) or OVA/Alum (100 μg/100 μg/mL) subcutaneously (s.c.) in the base of the tail. After 21 days, the mice groups received antigenic booster s.c. Group of non-immunized mice received 200 μL of water at the same route and was used as control.

### 2.8. Analysis of Plasma Cell and Memory B Cell Populations in Splenocyte Suspensions from Non-Immunized or Immunized Mice with OVA/Alum and OVA/PDDA-NPs

On day 28 after immunization, splenocyte suspensions were prepared from non-immunized or immunized mice. Spleens were collected and macerated in a sterile environment. After, red cells were lysed with a lysis buffer for 3 min at 4 °C, and the number and viability of the cells were determined using a Neubauer chamber and 0.1% Trypan Blue solution. The cell suspensions were incubated with Fcγ block mAb (1 μg/10^6^ cell) for 15 min at 4 °C. Afterwards, samples of 2 × 10^6^ cells diluted in 10.0 μL of PBS buffer were distributed in 96-well plates (Corning) and incubated with fixable viability staining (FVS) 510 (BD Biosciences) for 15 min at room temperature (RT). Subsequently, the cell samples were washed with phosphate buffer solution + 1% fetal bovine serum (PBS + 1% FBS) followed by centrifugation. The cells were resuspended and incubated with monoclonal antibodies, anti-surface markers of memory B cells (anti-CD19 PE, anti-CD27 BV421, and anti-CD38), and plasma cells (PCs) (anti-CD19 APC and anti-CD138 BV421).

Samples were acquired (50,000 events) by FACsCanto II and analyzed by FlowJo Software 10 (BD Bioscience, Franklin Lakes, NJ, USA). Parameters of size and granularity were selected, followed by “Single Cell” and selection of viable cells using FVS marking (FSC-A/AmCyan). From the viable cell population, the double-labeled CD19^+^CD138^+^ cell populations (plasma cells) were selected and, from the CD19^+^ population, the analysis of the double-labeled CD27^+^CD38^+^ population (memory B cells) was performed. For the analysis, the fluorescence-minus-one (FMO) methodology was used. Analyses were performed on samples obtained from individual animals (n = 4). Results were expressed as the mean of absolute number cells of individual animals/group ± standard deviation (SD) (Figure S1).

### 2.9. Analysis of the Expression of MHC Class II and Costimulatory Molecules in DCs from Cell Suspensions Obtained from Non-Immunized Mice or Mice Immunized with OVA/Alum or OVA/PDDA

Mice were immunized with OVA/Alum or OVA/PDDA. After 2, 3, and 4 days of immunization, cell suspensions were prepared from inguinal lymph nodes. Suspensions were resuspended in PBS + 1% FBS, distributed in 96-well plates (Corning), and incubated with anti-CD11c PE.Cy7, anti-MHC II FITC, and costimulatory labeled fluorophores: CD80 APC and CD40 BV421. Samples were fixed in PBS+ 1% formaldehyde (Merck). Samples were acquired in a FacsCanto II flow cytometer (BD Bioscience) and analyzed in FlowJo software. Analyses were performed on samples obtained from individual animals (n = 4–5). The analysis strategy was initiated by the parameters FSC-A x SSC-A, followed by the exclusion of clustered cells (FSC-A × FSC-H) and the selection of the population of live cells by marking with FVS. Then, the population of CD11c^+^ cells was analyzed and, from this cell population, the expression of molecules of MHC-II, CD40, CD80, and CD86 in mean fluorescence intensity (MIF) was analyzed. For the analysis, the fluorescence-minus-one (FMO) methodology was used. Results were expressed as the mean of absolute cell number of individual animals/group ± standard deviation (SD) or the MFI of MHC molecules or costimulatory expression of individual animals/group ± SD (Figure S2).

### 2.10. Differentiation of DCs from Bone Marrow (iBM-DCs) of BALB/c Mice

The femurs of BALB/c mice were collected using surgical kit in a sterile environment. The femoral cavity was washed with non-supplemented RPMI-1640 medium until complete removal of all bone marrow. The number and viability of the cells were determined using a Neubauer chamber and 0.1% Trypan Blue solution. Suspensions of 30 × 10^6^ cells were distributed in culture bottles in 20 mL in RPMI medium supplemented with 1% L-glutamine, 0.5% 2-mercaptoethanol, 1% penicillin-streptomycin, 1% vitamin, and 5% FBS plus 10 ng/mL of GM-CSF and 5 ng/mL of IL-4 and maintained at 5% CO_2_/37 °C. On day 4 of culture, the medium was replaced, and on the 7th day, the cells in suspension were collected and counted in the Neubauer chamber to carry out the in vitro experimental tests. The flow cytometer analysis of the cellular suspension showed 70–80% of CD11c^+^ cells.

### 2.11. In Vitro Experiment of iBM-DCs Stimulated with OVA, OVA/LPS, or OVA/PDDA-NPs

iBM-DCs (3 × 10^6^) were maintained in supplemented RPMI medium or incubated with OVA (100 µg/mL), OVA/PDDA (100/10 µg/mL), and OVA/LPS (100 µg/mL/250 ng/mL) for 18 h/37 °C. After this period, the cells were collected for analysis of MHC-II, CD40, CD80, and CD86 expression by flow cytometry. The culture supernatants were used for detection of IL-10, IL-12, and TNF-α by ELISA.

Suspensions of 1 × 10^6^/cells were labeled with the fixable viability stain (FVS) 510 (BD Biosciences) diluted in PBS for 15 min/R.T. After the FVS labeling, DC suspensions were stained for 30 min/4 °C with anti-CD80 APC, anti-CD86 PE, anti-MHC-II FITC, and anti-CD40 BV421 antibodies labeled with fluorophores; DCs were washed with PBS, centrifuged (1500 rpm/5 min/ 4 °C), and fixed with phosphate buffer solution (PBS) + 1% paraformaldehyde. The stained cells were read on the BD-FACsCanto II cytometer and analyzed on the FlowJo software. Initially, the analysis and selection of the total population was performed based on the parameters of granularity or internal complexity-side scatter area (SSC-A-on the y-axis) versus (vs) relative size and forward scatter area (FSC-A-on the y-axis) x), followed by the exclusion of debris and clustered cells through the analysis of FSC-H in relation to the area of FSC-A. Subsequently, the exclusion of the population of non-viable cells (marked with FVS) was performed, followed by the selection of the population of CD11c+ cells. The gated CD11c^+^ population was used for the analysis of the expression of MHC-II or costimulatory molecules. As a parameter to define the populations, the methodology of “fluorescence minus one” (FMO) was used, and the results were presented as the average of the fluorescence intensity of the costimulatory molecules or MHC II on the population of CD11c+ cells in the samples in triplicates ± SD (Figure S3).

### 2.12. Evaluation of TNF-α, IL-6, IL-12, and IL-10 Production by ELISA

The TNF-α production was measured using the eBioscience ELISA kit. IL-6 and IL-12 were evaluated by ELISA kits from R&D system. The IL-10 was detected by PEPROTECH ELISA Kit. All ELISA assays were performed according to the manufacturer’s protocols. The results were calculated by using a standard curve of the recombinant cytokine, and the data were expressed as the mean of the cytokine concentration of the samples in triplicate ± SD.

### 2.13. Flow Cytometry Analysis of Binding and Uptake of OVA/PDDA-NPs by iBM-DCs

In order to evaluate the binding of NPs to the DC membrane, as well as the internalization of the OVA/PDDA formulation, the conjugation of OVA-FITC (100 µg/mL) with PDDA (10 µg/mL) was carried out. Samples of BM-DCs (1 × 10^6^) were incubated with OVA-FITC/PDDA or OVA-FITC (100 µg/10 µg/mL) for 30 min at 4°or 37 °C, as described by Favoretto et al. (2017) [25]. All incubations were performed in triplicate. After this period, the cells were washed with PBS and resuspended in 200 µL of PBS with 1% paraformaldehyde. The reading was performed on the FACsCanto II Flow Cytometer and analyzed using the FlowJo software. Results were expressed as mean fluorescence intensity ± SD.

### 2.14. Antigen-Specific CD4T^+^ Lymphocyte Proliferation Assay

iBM-DCs were incubated with OVA (100 μg/mL), OVA/PDDA (100 μg/mL), or OVA/LPS (100 μg/mL/250 ng/mL) for 18 h/37 °C in 5% humidified CO_2_. Spleens of DO11.10 TCR transgenic mice specific for MHC-II–restricted OVA peptide were collected, macerated, and incubated with a lysis buffer (3 min/4 °C). After that, spleen cells were labeled with anti-CD4 PE.Cy-5 (15 min/R.T), filtered using cell strainer (Falcon, Thermo Fisher Scientific, Waltham, MA, USA), and purified by cell sorting (FACs Aria, BD Bioscience, Franklin Lakes, NJ, USA). Additionally, 0.6 × 10^5^ DCs were co-cultured with 3 × 10^5^ CD4^+^ T cells purified by cell sorting from DO11.10 TCR transgenic mice. To evaluate OVA-specific CD4^+^ T cell proliferation, the co-cultures were incubated for 48 or 72 h with BrdU (10 μM) in humidified 5% CO_2_ oven. Cell proliferation was evaluated using the BrdU-ELISA kit. All cultures were performed in quadruplicate, and results were expressed as mean OD ± SD.

### 2.15. Statistical Analysis

Flow cytometry analyses were presented by expression of molecules considering the mean fluorescence intensity (MFI) of triplicate samples ± standard deviation. Statistical analyses were performed using the one-way ANOVA test, followed by the Tukey method. Differences with *p* < 0.05, 0.001 and 0.0001 were considered statistically significant.

## 3. Results

### 3.1. OVA/PDDA Conjugation Produced a Colloidal Dispersion

**Figure 1:**
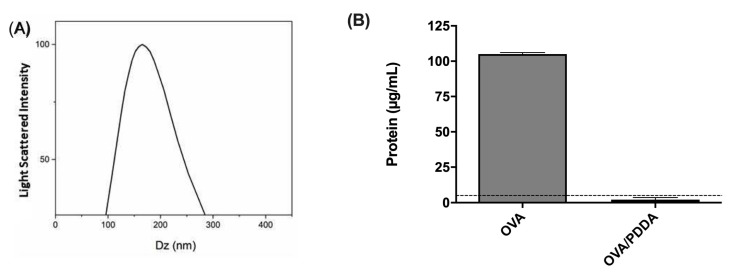
Characterization of OVA/PDDA-NPs. (**A**) Hydrodynamic diameter of NPs and (**B**) protein dosage; (**A**) After OVA-NPs formation, the hydrodynamic diameter was analyzed by Dynamic Light Scattering (DLS) in ZetaPlus Analyzer. (**B**) OVA/water (100 μg/mL) or OVA/PDDA (100 μg/10 μg/mL) samples were subjected to centrifugation at 14,500 rpm/30 min. Then, the supernatants were collected, and the protein concentration was determined by the BCA method. The dotted line represents the limit of detection from the BSA standard curve. The results are expressed as the mean of the protein content of samples in quadruplicate ± SD.

To evaluate the adjuvant effect of PDDA in OVA-specific immune response, OVA/PDDA-NPs were prepared, as previously described by Pérez-Betancourt et al., (2020) [24]. Figure 1 shows that the incubation of OVA/PDDA created a turbid colloidal dispersion of a peak of 178 nm (Figure 1A) in agreement with previously reported NPs size in OVA/PDDA water dispersions. Furthermore, after the centrifugation process, OVA was not detected in the supernatant of OVA/PDDA-NPs dispersions in contrast with the centrifuged OVA solution (Figure 1B).

### 3.2. OVA/PDDA Modulates OVA-Specific Adaptive Immune Response in Immunized Mice

**Figure 2:**
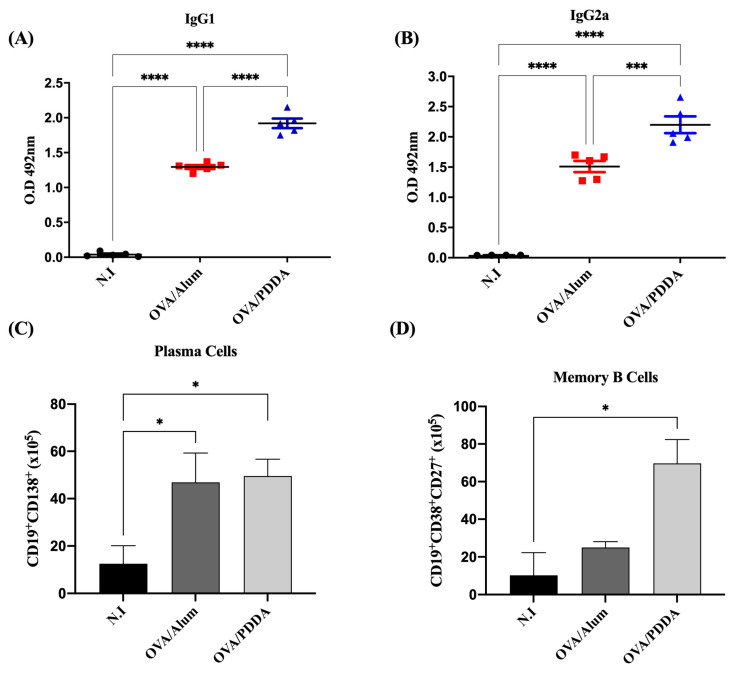
Potent effect of OVA/PDDA-NPs in induction of anti-OVA antibody production and B cell populations. Groups of BALB/c mice were immunized subcutaneously (s.c.) with 200 μL of OVA/Alum (100 μg/100 μg/mL) or OVA/PDDA (100 μg/10 μg/mL). As a control, mice received 200 μL of water. On the 21st day post-immunization, the mice received an antigenic booster, and on day 28, they were bled for antibody evaluation by ELISA. Their spleens were also collected to analyze the plasma cell (CD19^+^CD138^+^) and memory B (CD19^+^CD27^+^CD38^+^) cell populations by flow cytometry, as described in Materials and Methods. (**A**) Anti-OVA IgG1 (1/1280) and (**B**) anti-OVA IgG2a (1/20) antibodies in samples of individual mice/group. The results represent the optical density of the mean of the samples (n = 4) ± SD. (**C**) Number of plasma cells and (**D**) memory B cells in splenocyte suspensions of the individual mice/group (n = 4–5)/group ± SD. * *p* < 0.01 of significance; *** *p* < 0.001 of significance, **** *p* < 0.0001 of significance.

Pérez-Betancourt et al. (2020) demonstrated that OVA/PDDA-NPs induced higher DTH and anti-OVA antibody responses when compared with the OVA/Alum group [24]. Our data also showed robust anti-OVA IgG1 and IgG2a antibody production in OVA/PDDA-immunized mice compared with the OVA/Alum group (Figure 2A,B). Consistently, there was an increase in plasma cells (CD19^+^CD138^+^) in mice immunized with OVA/PDDA or OVA/Alum (Figure 2C). Reinforcing the potent adjuvanticity of OVA/PDDA-NPs, a higher number of memory B cells (CD19^+^CD38^+^CD27^+^) in the OVA/PDDA group was observed in comparison with the OVA/Alum or control groups (N.I.) (Figure 2D).

### 3.3. OVA/PDDA-NPs Immunization Increases the Number of CD11c^+^ Cells Expressing Molecules Involved in Antigen Presentation in Draining Lymphoid Tissue of Mice

**Figure 3:**
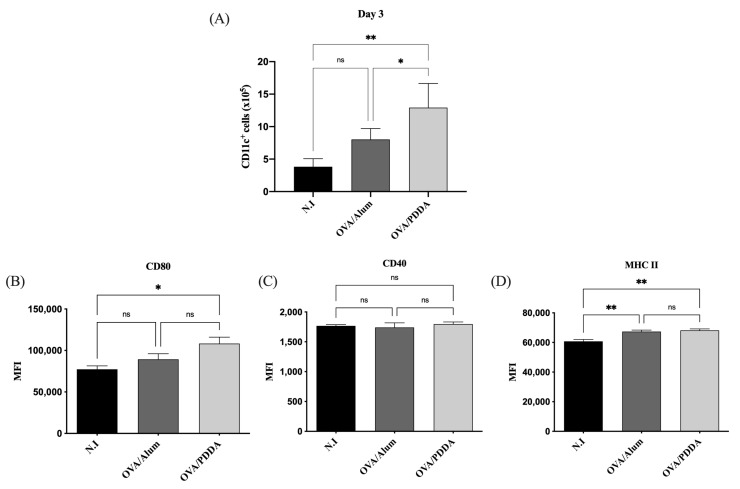
Effect of OVA/PDDA immunization in migration of CD11c^+^ cells to draining lymph nodes and DCs maturation in vivo. Groups of BALB/c mice were immunized (s.c.) with 200 μL of OVA/Alum (100 μg/100 μg/mL) or OVA/PDDA (100 μg/10 μg/mL). As a control, mice received 200 μL of water. After 3 days of immunization, inguinal lymph nodes were obtained, and 1 × 10^6^ cells were incubated with anti-CD11c, anti-CD80, anti-CD40, and anti-MHC II mAb labeled with fluorophores and analyzed by flow cytometry. (**A**) Number of CD11c^+^ cells from mice groups immunized 3 days before; expression of (**B**) CD80, (**C**) CD40, and (**D**) MHC-II molecules on CD11c^+^ cell population from mice groups immunized 3 days before. The analysis strategy is described in material and methods. The results represent the mean of the number of CD11c^+^ cells from lymph node cell suspensions of individual mice/group (n = 4) ± SD. Mean of fluorescence intensity (MFI) of molecule expression on CD11c+ cells from individual mice/group (n = 4) ± SD. Statistical analysis was performed by one-way ANOVA with Tukey’s post-test. * *p* < 0.01 of significance; ** *p* < 0.05 of significance. Representative results from three independent experiments.

DCs have been considered the link between the innate and the adaptive immune response [20]. Therefore, we analyzed this cell population on draining lymph nodes on the first days of OVA/PDDA-NPs immunization. After the first 2 days of immunization, we did not verify DC migration on draining tissue of OVA/PDDA-NP or OVA/Alum groups compared with non-immunized mice. However, on the 3rd day, there was an increase in CD11c^+^ cells in the lymph nodes of the OVA/PDDA-NPs group compared with non-immunized (N.I.) or OVA/Alum mice, as shown in Figure 3A. In addition, the upregulation of CD80 and MHC-II expression on CD11c^+^ cells from OVA/PDDA mice was observed compared to the control group. The higher expression of MHC-II molecules on CD11c^+^ cells of the OVA/Alum group was also observed (Figure 3B–D).

**Figure 4:**
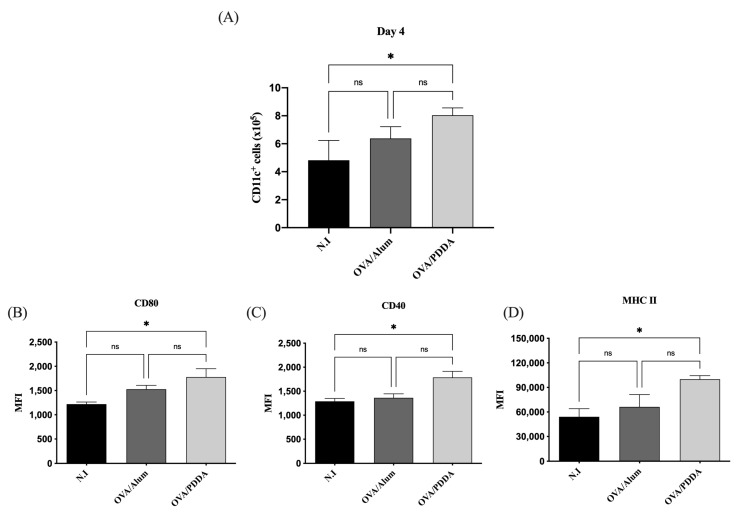
Migration of CD11c^+^ cells to draining lymph nodes induced by OVA/PDDA immunization 4 days before. Groups of BALB/c mice were immunized subcutaneously (s.c.) with 200 μL of OVA/Alum (100 μg/100 μg/mL) or OVA/PDDA (100 μg/10 μg/mL). As a control, mice received 200 μL of water. After 4 days of immunization, inguinal lymph nodes were obtained, and 1 × 10^6^ cells were incubated with anti-CD11c, anti-CD80, anti-CD40, and anti-MHC II mAbs labeled with fluorophores and analyzed by flow cytometry. (**A**) Number of CD11c^+^ cells from mice groups immunized 4 days before. Expression of (**B**) CD80, (**C**) CD40, and (**D**) MHC-II molecules on CD11c^+^ cell population from mice groups immunized 4 days before. The analysis strategy is described in material and methods. The results represent the mean of the number of CD11c^+^ cells from lymph node cell suspensions of individual mice/group (n = 4) ± SD. Mean of fluorescence intensity (MFI) of molecule expression on CD11c+ cells from individual mice/group (n = 4) ± SD. Statistical analysis was performed by one-way ANOVA with Tukey’s post-test. * *p* < 0.01 of significance. Representative results from three independent experiments.

Four days after mice immunization, the population of CD11c^+^ cells was increased in draining lymph nodes of the OVA/PDDA group accompanied by the increased expression of costimulatory and MHC-II molecules on these cells (Figure 4A–D).

### 3.4. OVA/PDDA-NPs Did Not Exert Cytotoxicity on iBM-DCs in Culture

**Figure 5:**
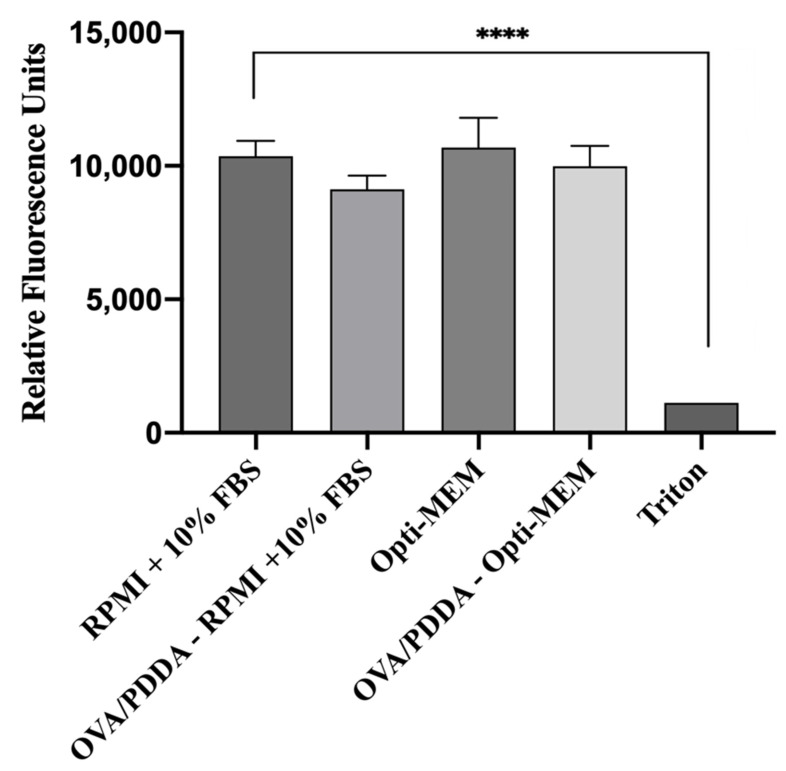
Effect of OVA/PDDA-NPs on viability of BM-DCs in culture. The Presto Blue assay was performed in iBM-DCs cultures incubated with OVA/PDDA-NPs for 24 h in RPMI medium supplemented with 10% FBS or OPT-MEM. Fluorescence was determined by the means of the samples in quadruplicate ± SD. **** *p* < 0.0001 of significance.

Cationic polymers are known to promote adose-dependent cytotoxicity [25,26,27]; therefore, the viability of immature bone marrow-DCs (iDCs) cultured in vitro with OVA/PDDA-NPs (100 μg/10 μg/mL) was evaluated. For this, cultures of iDCs in RPMI medium supplemented with 10%FBS or OPT-MEM medium were incubated with OVA/PDDA-NPs for 24 h, according to Pérez-Betancourt et al. (2020) [24]. The data showed that OVA/PDDA-NPs did not exert cytotoxicity on iDCs (Figure 5).

### 3.5. Binding and Uptake of OVA/PDDA-NPs by BM-DCs In Vitro

**Figure 6:**
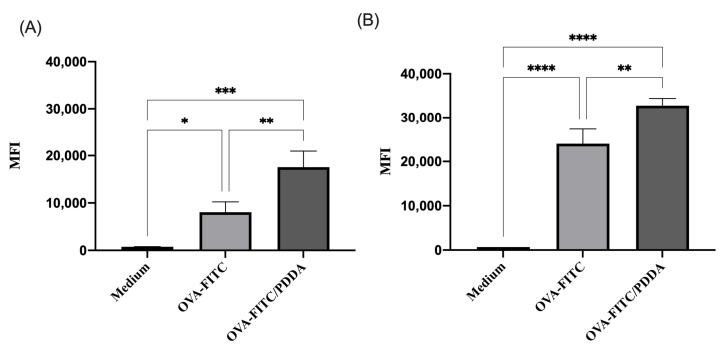
Binding and uptake of OVA-FITC/PDDA and OVA-FITC by iBM-DCs. (**A**) iBM-DCs were incubated with OVA-FITC or OVA-FITC/PDDA for 30 min at 4 °C and (**B**) at 37 °C. The samples were analyzed by flow cytometry. Results are expressed as mean fluorescence intensity (MIF) of the samples in triplicate ± SD. * *p* < 0.01 of significance; ** *p* < 0.05 of significance; *** *p *< 0.001 of significa and **** *p* < 0.0001 of significance. Representative results of three independent experiments.

Aiming to understand the potential effect of OVA/PDDA on antigen-presenting cells (APCs), the binding and uptake of NPs by iBM-DCs was determined using OVA-FITC and OVA-FITC/PDDA-NPs incubation at 4° and 37 °C, followed by an analysis by flow cytometry. The fluorescence intensity of DCs incubated with OVA-FITC/PDDA was higher than the one of DCs incubated with OVA-FITC at 4 °C (Figure 6A) of NP binding. Similar results were obtained in iBM-DCs incubated with OVA-FITC or OVA/PDDA-FITC at 37 °C (Figure 6B).

### 3.6. OVA/PDDA-NPs Induced BM-DCs Maturation In Vitro

**Figure 7:**
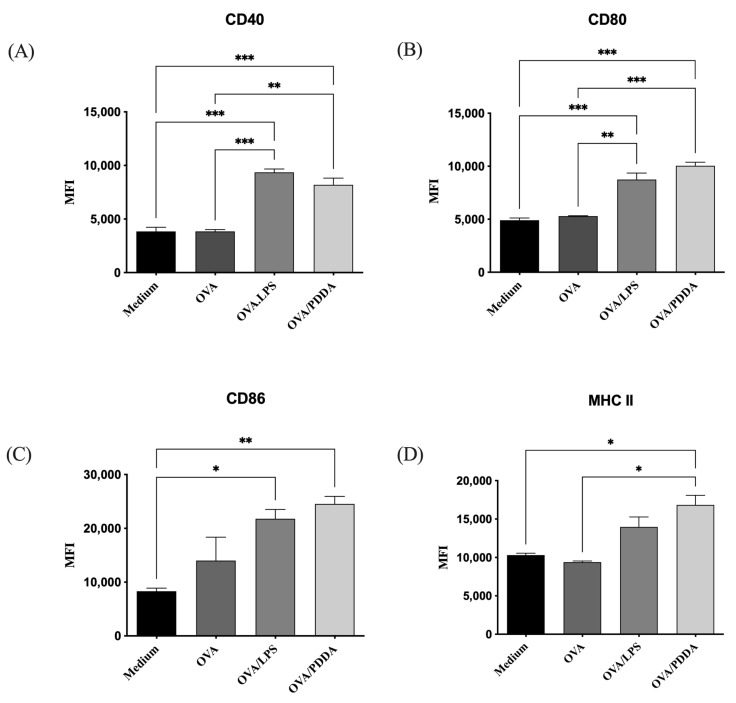
Effect of OVA/PDDA-NPs in the expression of costimulatory and MHC-II on DC cultures. iBM-DCs (1 × 10^6^) were incubated for 18 h with OVA/LPS, OVA, and OVA/PDDA-NPs. After this, the DCs were incubated with anti-CD11c, anti-CD80, anti-CD86, anti-CD40, and anti-MHC II mAbs labeled with fluorophores and analyzed by flow cytometry. The analysis strategy is described in Supplementary Material. The results represent the mean of fluorescence intensity of (**A**) CD40, (**B**) CD80, (**C**) CD86 and (**D**) MHC II expression on CD11c^+^ cells in triplicate ± SD. * *p* < 0.05 of significance; ** *p* < 0.01 of significance; *** *p* < 0.001 of significance. Representative results from three independent experiments.

Considering that the binding and uptake of different antigens are crucial for the DC maturation and antigen presentation [28], iBM-DCs were incubated with OVA, OVA/PDDA, or OVA/LPS for 18 h at 37 °C. Afterward, the ability of OVA/PDDA-NPs to induce iBM-DC maturation was analyzed by flow cytometry. OVA/PDDA-NPs upregulated the expression of CD80, CD86, CD40, and MHC-II on DCs, similarly to OVA/lipopolysaccharides (LPS) (Figure 7).

### 3.7. OVA/PDDA-NPs Induced Production of Pro-Inflammatory Cytokines by BM-DCs

**Figure 8:**
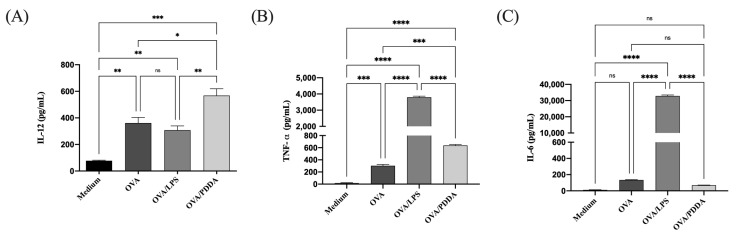
Cytokine production by DCs incubated with OVA/PDDA in vitro. iBM-DCs (1 × 10^6^) were incubated with OVA, OVA/LPS, or OVA/PDDA for 18 h. Then, the cell supernatants were collected to detect the cytokines using ELIS (**A**) IL-12, (**B**) TNF-alpha and (**C**) IL-6 production. The results represent the mean of the cytokine production in the samples in triplicate ± SD. * *p* < 0.05; ** *p* < 0.01; *** *p* < 0.001 and **** *p* < 0.0001 of significance. Representative results from two independent experiments.

The production of TNF-α and IL-12 was higher in supernatants of DCs incubated with OVA/PDDA-NPs, OVA, or OVA/LPS than in DCs incubated with medium only (Figure 8). However, OVA-LPS predominantly induced the production of TNF-α by DCs, whereas OVA/PDDA increased IL-12 production. Only OVA/LPS stimulated IL-6 production by DCs. IL-10 was not detected by the ELISA assay. Therefore, the production of proinflammatory cytokines and the high expression of co-stimulatory molecules and MHC-II by DCs are strong evidence that OVA/PDDA-NPs are able to induce DC maturation.

### 3.8. High OVA-Specific CD4^+^T Cell Proliferation Induced by BM-DCs Incubated with OVA/PDDA-NPs

Since OVA/PDDA-NPs were able to induce DC maturation in vitro, we evaluated the ability of these DCs to promote the proliferation of OVA-specific CD4^+^T cells. For this, iBM-DCs were incubated with RPMI medium (control), OVA, OVA/LPS, or OVA/PDDA for 18 h. Afterward, the DCs were co-cultured with CD4^+^T cells purified from DO11.10 TCR transgenic mice specific for MHC-II–restricted OVA peptide for 72 h.

**Figure 9:**
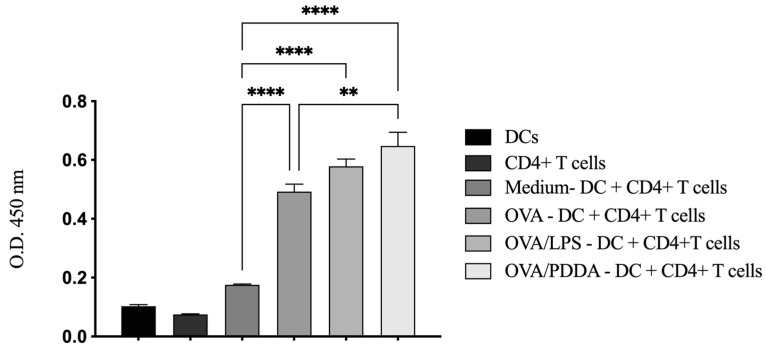
DCs incubated with OVA/PDDA-NPs are able to induce proliferation of OVA-specific CD4+T cells in vitro. iDCs differentiated in vitro were incubated with OVA, OVA/LPS, and OVA/PDDA for 18 h. After this, the DCs were co-cultured with CD4^+^ T cells purified from DO11.10 mice for 72 h. The proliferative response was evaluated using BrdU-ELISA assay. Results expressed as the mean of optical density obtained in samples in quadruplicate ± SD. ** *p* < 0.01 and **** *p* < 0.0001 of significance.

The results showed that DCs incubated with OVA, OVA/PDDA, or OVA/LPS induced a proliferative response of OVA-specific T cells when compared with co-cultures of DCs plus CD4+T cells maintained in the culture medium. Furthermore, the proliferative response of CD4+T cells induced by DCs previously incubated with OVA/PDDA was higher than those elicited from co-cultures of CD4^+^T cells with DCs incubated with OVA (Figure 9).

## 4. Discussion

Pérez-Betancourt et al. (2020) showed that nanoparticles of the cationic polymer poly (diallyl dimethyl ammonium chloride) (PDDA) entangled with OVA elicited high anti-OVA antibody and cellular immune responses in a murine model of immunization [29,30]. Therefore, here, we clarify the mechanism involved in the induction of antigen-specific immune response elicited by OVA/PDDA-NPs as mediated by DCs. The physical properties of OVA/PDDA-NPs such as size corroborated those described by Pérez-Betancourt et al. (2020) [24]. In addition, no detectable OVA in the supernatant of ultra-centrifuged OVA/PDDA dispersions reconfirmed the existence of the OVA/PDDA-NPs in the pellet.

The present data also demonstrated that OVA/PDDA-NPs induced high production of OVA-specific IgG1 and IgG2a, as described by Pérez-Betancourt et al. (2020) [24]. Furthermore, here, the data add further information on cell populations in mice elicited by OVA/PDDA-NPs, namely an increased number of CD19^+^CD138^+^ cells and CD19^+^CD38^+^CD27^+^ memory B cells in mice. The generation of memory B cells that are primed to react faster for the antigen/pathogen, with greater intensity and high affinity than in the primary immune response is an important result described in this work ensuring long-term vaccine efficacy [31,32]. As is known, antigen/pathogen-experienced memory B cells represent the second line of defense against homologous challenges that are rapidly reactivated to produce antibodies mediating immune protection [33,34]. Our data showed that OVA/PDDA-NPs elicited a number of memory B cells (CD19^+^CD38^+^CD27^+^ cells) even higher than the one elicited by OVA/Alum immunization, demonstrating the potent effect of the NPs in the induction of a robust antigen-specific immunity. Furthermore, the significant increase in plasma cells (CD19^+^CD138^+^ cells) in the OVA/PDDA-NPs mice group is in agreement with the high levels of anti-OVA antibody production detected in our experiments. Importantly, OVA/PDDA-NPs were more efficient in generating memory B cell response in comparison with OVA/Alum. In fact, nanostructures studied in vaccine design are able to modulate immune response [35,36,37], and the role of innate immunity has been correlated with the efficiency of vaccines to promote an appropriate protective immune response [38].

DCs have a functional plasticity participating in either adaptive immune induction or tolerance process [39]. In an immature state, DCs are situated in the periphery, strategically recognizing and capturing antigens. Upon antigen stimulation, DCs migrate to lymphoid-draining tissue and present processed peptides derived from captured antigens complexed with major histocompatibility complex (MHC) and costimulatory molecules, and these cells also secrete cytokines to promote T cell activation and differentiation [40]. The analysis of the CD11c^+^ cells in our murine immunization model showed a higher number of this cell population induced by OVA/PDDA-NPs in comparison with naïve mice. Furthermore, CD11c^+^ cells with upregulated expression of MHC-II and costimulatory molecules were significantly induced by OVA/PDDA-NPs, similar to gold-NPs (Au-NPs) as a vaccine delivery agent with distinct antigens [39]. Therefore, the results demonstrate the ability of OVA/PDDA-NPs to upregulate the DC maturation and migration to lymphoid tissue, clarifying how OVA/PDDA-NPs activated innate immunity. Furthermore, the data demonstrated the uptake of OVA/PDDA dispersions by DCs and also its ability to potentiate the functional activity of DCs to induce OVA-specific CD4^+^T cell proliferation.

In in vitro assays using BM-DCs, the conjugation of OVA with PDDA promoted an increase in the binding and uptake of OVA/PDDA-NPs by iBM-DCs compared with OVA solution, as depicted from fluorescence-labeled OVA analyzed by flow cytometry. Different cellular entry routes are available for nanoparticles during in vivo and in vitro cell exposure. Some evidence reinforces that cationic NPs bind to lipid bilayers and can enter cells by endocytosis or direct translocation; however, the exact way NPs gain access into cells is under investigation [41,42]. In agreement with the present results, Chang and colleagues (2017) [37] previously demonstrated, using flow cytometry and microscopy, that OVA/PDDA-NPs of different sizes were more internalized by DCs when compared to soluble OVA.

In addition, the ability of the OVA/PDDA-NPs’ immunization to promote the migration and maturation process of DCs to the lymphoid tissues of mice was verified. Furthermore, the data also showed that OVA/PDDA-NPs promoted the enhancement of DCs expressing MHC-class II and molecules involved in antigen-presenting, as well as the cytokine secretion in vitro, which mediate CD4^+^T cell activation and differentiation for effector cells. DCs previously incubated with OVA/PDDA-NPs also induced the highest CD4^+^T cell proliferation when compared with DCs incubated with OVA/LPS or soluble OVA in vitro.

In accordance with our data, Xu et al. (2012), studying gold nanostructures of cetyltrimethylammonium bromide (CTAB), poly(diallyl dimethyl ammonium chloride) (PDDA), and polyethyleneimine (PEI) combined with HIV-1 Env plasmid DNA (Env), showed the ability of PDDAC-Au NR, PDDAC-Au NR-Env complex, PEI-Au NR, or the PEI-Au NR-Env complex, but not CTAB-Au NR or CTAB-Au NR-Env, to induce mature DCs (CD11c^+^MHCII^+^CD86^+^CD80^+^ cells) in vitro [43].

Versatile vaccine adjuvants should include memory immunity as our OVA/PDDA-NPs do. Our results showed the adjuvant potential of OVA/PDDA-NPs to induce in mice OVA-specific immune response by promoting the migration of CD11c^+^ cells with mature phenotype. In addition, OVA/PDDA-NPs promoted increased expression molecules involved in antigen presentation by DCs, as well as the production of IL-12 and TNF-α. Last but not least, BM-DCs incubated with OVA/PDDA-NPs were able to induce proliferation of OVA-specific CD4^+^T lymphocytes in vitro.

Taken together, the data demonstrate the ability of cationic OVA/PDDA-NPs, as an adjuvant to protein molecules, to induce a robust adaptive immune response. Corroborating Pérez-Betancourt et al. (2020), our data demonstrate the potential effect of PDDA to promote protein antigen immune response, improving innate immunity activation and consequent adaptive immune response [24]. DCs are the cell type responsible for adaptive immunity induction, and their activation and maturation are required for CD4^+^ cell response. The binding and uptake increase could be the key for PDDA’s adjuvant role. In this sense, improvement of DC uptake and binding is an action mechanism reported for different adjuvants [44,45,46]. The binding can trigger pathways mediated by membrane surface receptors on DCs and consequently the cell maturation. Mature DCs are able to induce T lymphocyte activation and different effector profiles, such as Th1 and Th2 [47]. IgG1 and IgG2a produced by B cells are dependent on the cytokines secreted by Th2 and Th1 profile immune responses. Our results provide evidence on the important ability of PDDA/antigen NPs to strongly elicit antibody-producing B cells.

Cationic nanocarriers have been extensively studied as adjuvants for antigenic structures. Lei et al. (2020) evaluated the ability of chitosan, PEI, DOTAP, and the liposome (NeutralL) as adjuvants for SARS-CoV-2 in murine model, and the results showed an enhanced immunity in intranasal and intramuscular immunization. The data also showed that the intranasal immunization with PEI promoted higher production of IgG1, IgG2a, and IgG2b in mice when compared with the other adjuvants and control groups, while the increased production of antibodies was induced by all cationic nanocarriers administered by the intramuscular route. On murine DCs, PEI increased the uptake of antigens by DCs with the release of proinflammatory cytokines. In anti-tumoral vaccines, PEI induced DC activation with high IL-12 production [48]. Jin and collaborators (2022) used PEI as an adjuvant for antifungal vaccines, also showing B cell differentiation for long-lived plasma cells (LLPC) on C57BL/6 mice immunization [49].

In this context, our results show the dynamic of the innate immunity activation targeted by the OVA/PDDA-NPs immunization promoting the maturation of DCs with migration to lymphoid tissue. Using the optimizing formulation of OVA/PDDA-NPs, we achieved a system with high biocompatibility while maintaining robust immunostimulatory properties. This is particularly relevant, as safety concerns have limited the broader application of cationic materials in vaccine development. Our data show that OVA/PDDA-NPs do not exert cytotoxic effects on DCs, providing a promising balance between efficacy and safety.

Furthermore, the data demonstrated the increased uptake of OVA/PDDA-NPs by DCs and the ability to potentiate the functional activity of DCs to induce OVA-specific CD4^+^T cell proliferation as a component of an adaptive immune response. The robust adaptive immune response induced by OVA-PDDA NPs culminated in a significantly increased number of plasma cells and memory B cells in mice.

## 5. Conclusions

The data presented here show that OVA/PDDA-NPs immunization safely activates innate immunity promoting the DC maturation and induction of a robust adaptive immune response, indicating that this cationic material is an interesting approach for vaccine development.

## Data Availability

The original contributions presented in this study are included in this article; further inquiries can be directed to the corresponding author.

## Supplementary Materials

The following supporting information can be downloaded at: https://www.mdpi.com/article/10.3390/vaccines13010076/s1, Figure S1: Analysis of in vivo B cell populations; Figure S2: Analysis of in vivo CD11c+ cells; Figure S3: Analysis of in vitro CD11c+ cells.
