# Supplementary figures and images for: The Role of Dendritic Cells in Adaptive Immune Response Induced by OVA/PDDA Nanoparticles
Source: Vaccines (Basel). 2025 Jan 16;13(1):76. doi: 10.3390/vaccines13010076 (PMC11769024; doi:10.3390/vaccines13010076)

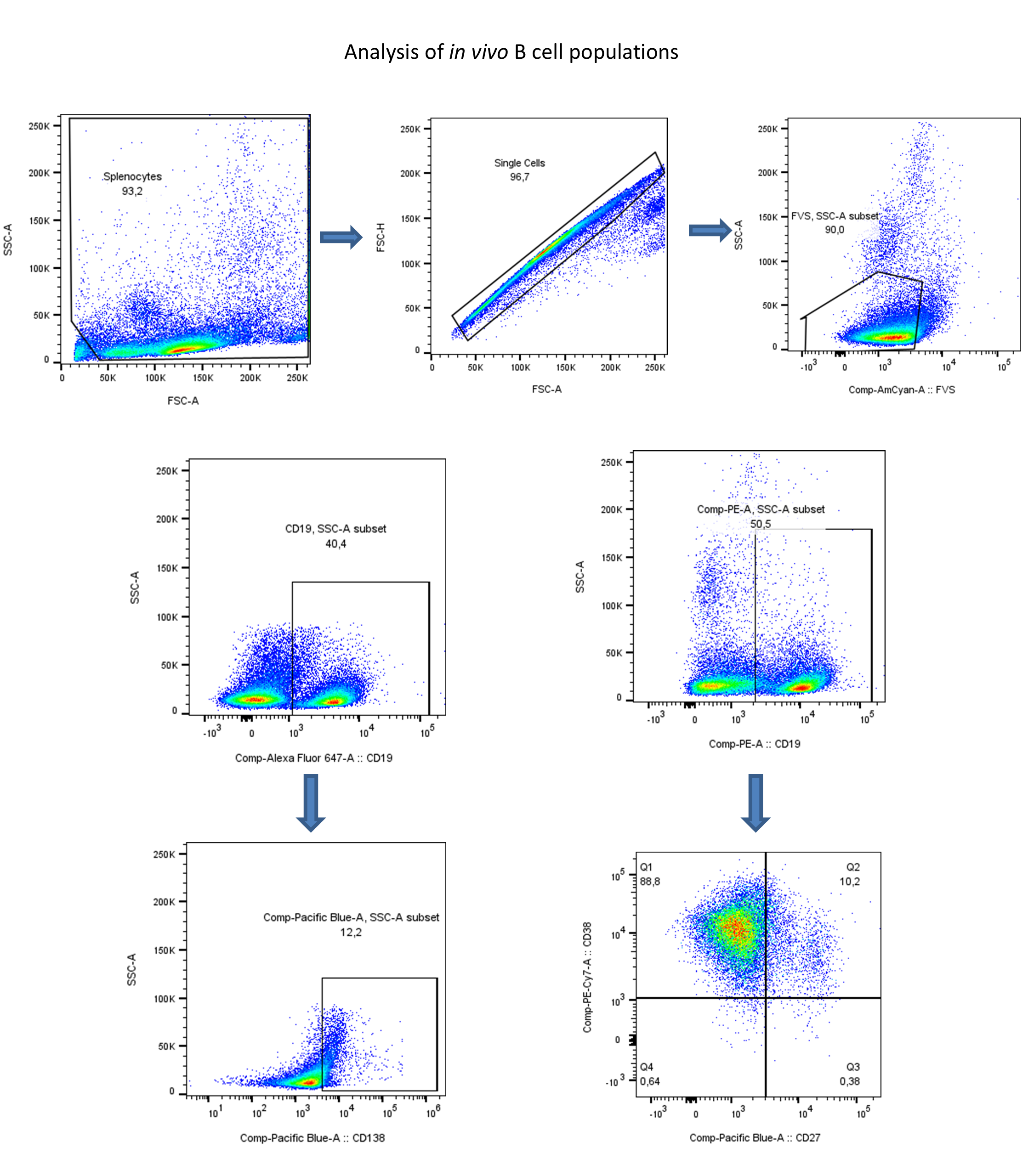

Supplement: Supplementary file 1 [file vaccines-13-00076-s001.zip › Figure S1. Analysis of in vivo B cell populations.tif]

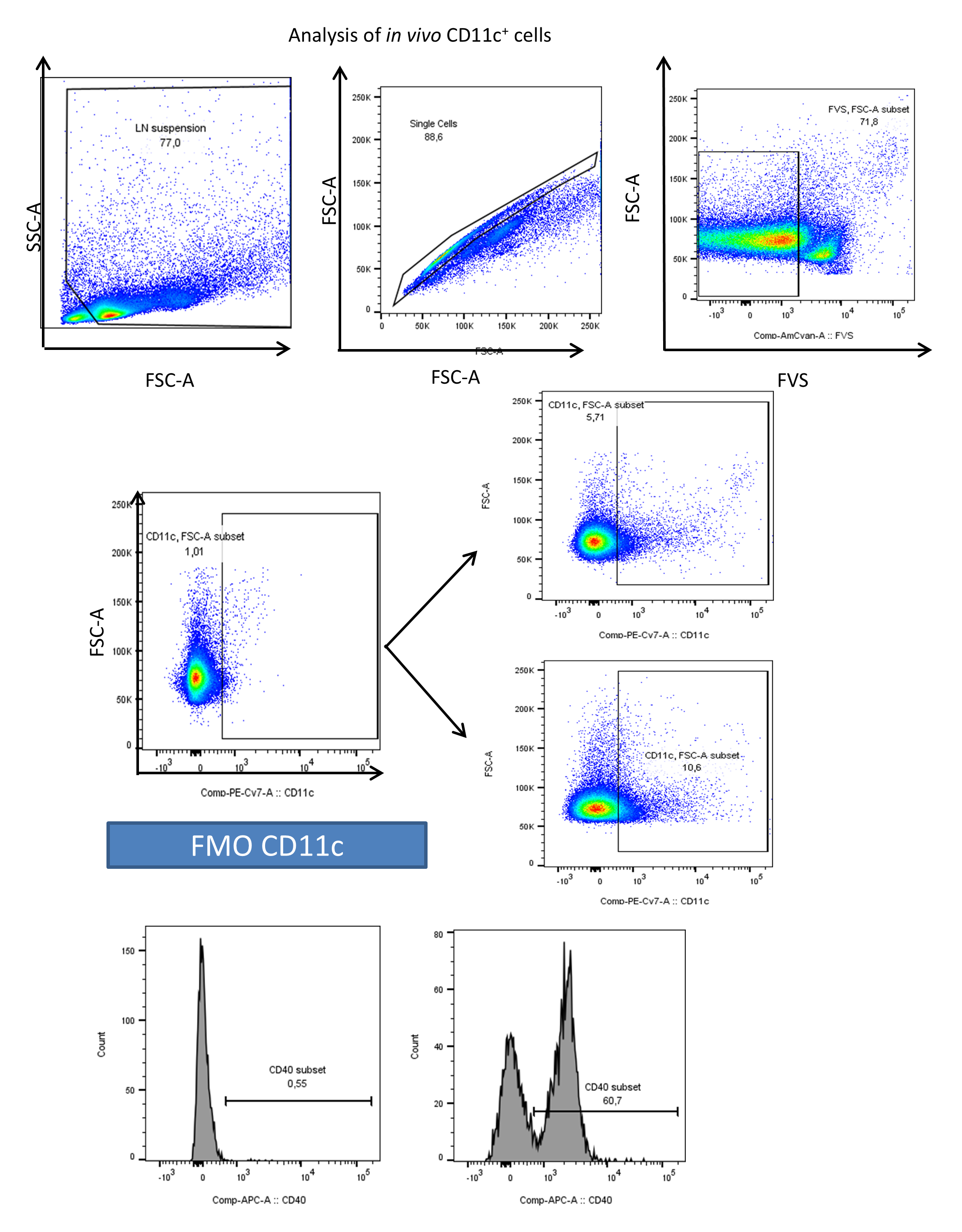

Supplement: Supplementary file 1 [file vaccines-13-00076-s001.zip › Figure S2. Analysis of in vivo CD11c+ cells.tif]

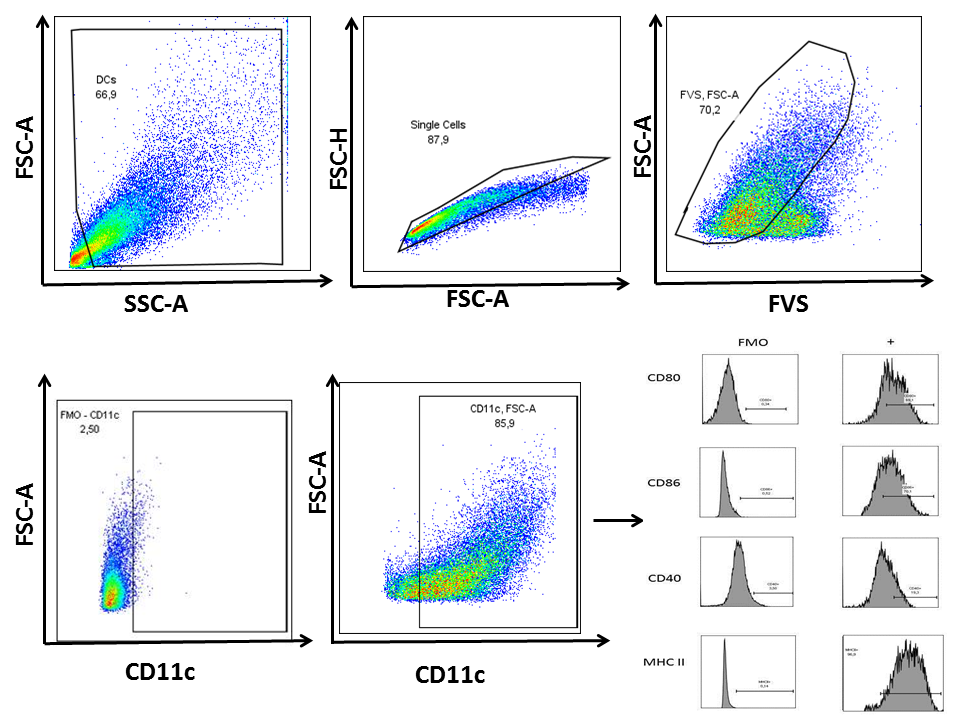

Supplement: Supplementary file 1 [file vaccines-13-00076-s001.zip › Figure S3. Analysis of in vitro CD11c+ cells.tif]
